# Gut microbial signatures are associated with Lynch syndrome (LS) and cancer history in Druze communities in Israel

**DOI:** 10.1038/s41598-023-47723-3

**Published:** 2023-11-24

**Authors:** Rawi Naddaf, Shaqed Carasso, Gili Reznick-Levi, Erez Hasnis, Amalfi Qarawani, Itay Maza, Tal Gefen, Elizabeth Emily Half, Naama Geva-Zatorsky

**Affiliations:** 1 Technion Israel Institute of Technology the Ruth and Bruce Rappaport Faculty of Medicine, Haifa, Israel; 2 Rappaport Technion Integrated Cancer Center, Haifa, Israel; 3 Genetics Institute Rambam Health Care Campus, Haifa, Israel; 4 Gastroenterology Institute Rambam Health Care Campus, Haifa, Israel; 5 Canadian Institute for Advanced Research, Toronto, ON Canada

**Keywords:** Cancer screening, Gastrointestinal cancer, Tumour biomarkers, Cancer, Microbial communities

## Abstract

Lynch syndrome (LS) is a hereditary cancer syndrome caused by autosomal dominant mutations, with high probability of early onset for several cancers, mainly colorectal cancer (CRC). The gut microbiome was shown to be influenced by host genetics and to be altered during cancer development. Therefore, we aimed to determine alterations in gut microbiome compositions of LS patients with and without cancer. We performed fecal microbiome analyses on samples of LS and non-LS members from the Druze ethnoreligious community in Israel, based on both their LS mutation and their cancer history. Our analysis revealed specific bacterial operational taxonomic units (OTUs) overrepresented in LS individuals as well as bacterial OTUs differentiating between the LS individuals with a history of cancer. The identified OTUs align with previous studies either correlating them to pro-inflammatory functions, which can predispose to cancer, or to the cancer itself, and as such, these bacteria can be considered as future therapeutic targets.

## Introduction

The human gut microbiome has been associated with a variety of host conditions in health and disease. Specifically, the gut bacterial composition was shown to be altered in various disease states, and to affect host physiology, immune system, and diseases^1–6^. Studies have demonstrated correlations between microbiome composition and tumor aggressiveness and management, in colorectal cancer (CRC) and other types of cancers^4,7–12^. In parallel, studies have shown that different genetic backgrounds of the host, (e.g., mutations) can also affect microbiome composition^13–15^. Altogether, these highlight the interest in studying microbiome compositions in heredity conditions associated with gastro-intestinal (GI) tumors as well as other types of cancer. Such microbiome alterations can potentially lead to understanding causality, improved diagnostic tools, and can affect treatment strategies.

Lynch syndrome (LS) is one of the most common hereditary cancer syndromes with a calculated estimated frequency of approximately 1:350 in western countries^16^. LS is characterized by early onset of a diverse spectrum of cancers (mainly CRC and endometrial cancer), and accounts for 3–5% of all incidents of CRC as well as endometrial cancer worldwide^17^. It is an autosomal dominant disorder, caused by a mutation in one of 4 mismatch repair (MMR) genes namely MLH1, MSH2, MSH6 and PMS2^18,19^. Carriers of specific MMR gene mutations show sex- and age-related patterns as well as a large phenotypic diversity of cancer risk and survival^17,20^. This phenomenon is regarded as multifactorial with contribution of both genetic and environmental factors.

The Druze is considered an ethnoreligious Middle Eastern minority group. The Druze worldwide population is estimated to be about 1.2 million who reside mainly in Syria (50%), Lebanon (30%), and to a lesser extent in Jordan, USA, Saudi Arabia and Israel. As of 2021 about 149,000 Druze reside in Israel according the Israeli Central Bureau of Statistics (Reported on April 24th, 2022). As part of their religion the Druze have adhered to marriages only within their community. In addition, one can only be born Druze—it is not possible to convert to a Druze from an outside community. The Druze in Israel reside in a limited number of villages mainly in the north of the country.

To the best of our knowledge the only LS causing mutation among Druze LS families has been a deletion in exon 4 at position 705 causing an early stop codon in MSH2 gene; c.705del (p.Asp236fs) which was originally described by Zidan and Friedman in 2008^21^. Taken together the fact that the Druze are a unique genetic population who share similar environments (i.e., geographical, household, dietary) makes them an ideal population to study phenotypic diversity.

Since the microbiome is largely affected by the environment, we hypothesize that analyzing the microbiome composition in the Druze population, in regards to LS genetic predisposition, can be key to our understanding of cancer-microbiome associations.

The gut microbiome was studied before in the context of LS. One study examined fecal and colon tissue microbiome composition of 100 individuals with several different mutations leading to LS and identified correlations to the development of adenomas^22^. Another study examined the fecal microbiome compositions of 8 LS individuals, by 16S sequencing, and correlated them with their cancer status^23^. The differences identified in this study were non-significant, which could be explained by their small sample size and low-resolution sequencing methods. An additional study examined the fecal microbiome composition of 10 female LS individuals with CRCs, endometrial tumors, or both comparing them to 8 non-LS female individuals^24^. Although this study did not detect microbiome alterations based on cancer type, they were successful in finding differences between LS individuals and the healthy controls, which indicates a possible connection between the LS genetic background and the fecal microbiome.

Here we present a different approach. We examined the fecal microbiome composition of individuals from the specific, Druze community, comparing LS to non-LS individuals. The LS individuals carried the same mutation in MSH2 (with or without cancer medical history), while the non-LS individuals were family members of the LS carriers in the study, some residing in the same household. This is especially important since person-to-person microbiome transmissions are becoming increasingly evident^25,26^. Including family members as controls enabled meticulous microbiome-based analysis within the cohort with the genetic mutational status as the focal point of divergence, while minimizing the environmental impact on microbial diversity. We performed shotgun metagenomics sequencing, comparing the microbiome composition between LS and non-LS participants and within the LS participants we correlated the microbiome composition in regards to their cancer history records. In this report we provide analysis of the results.

## Results

**Figure 1:**
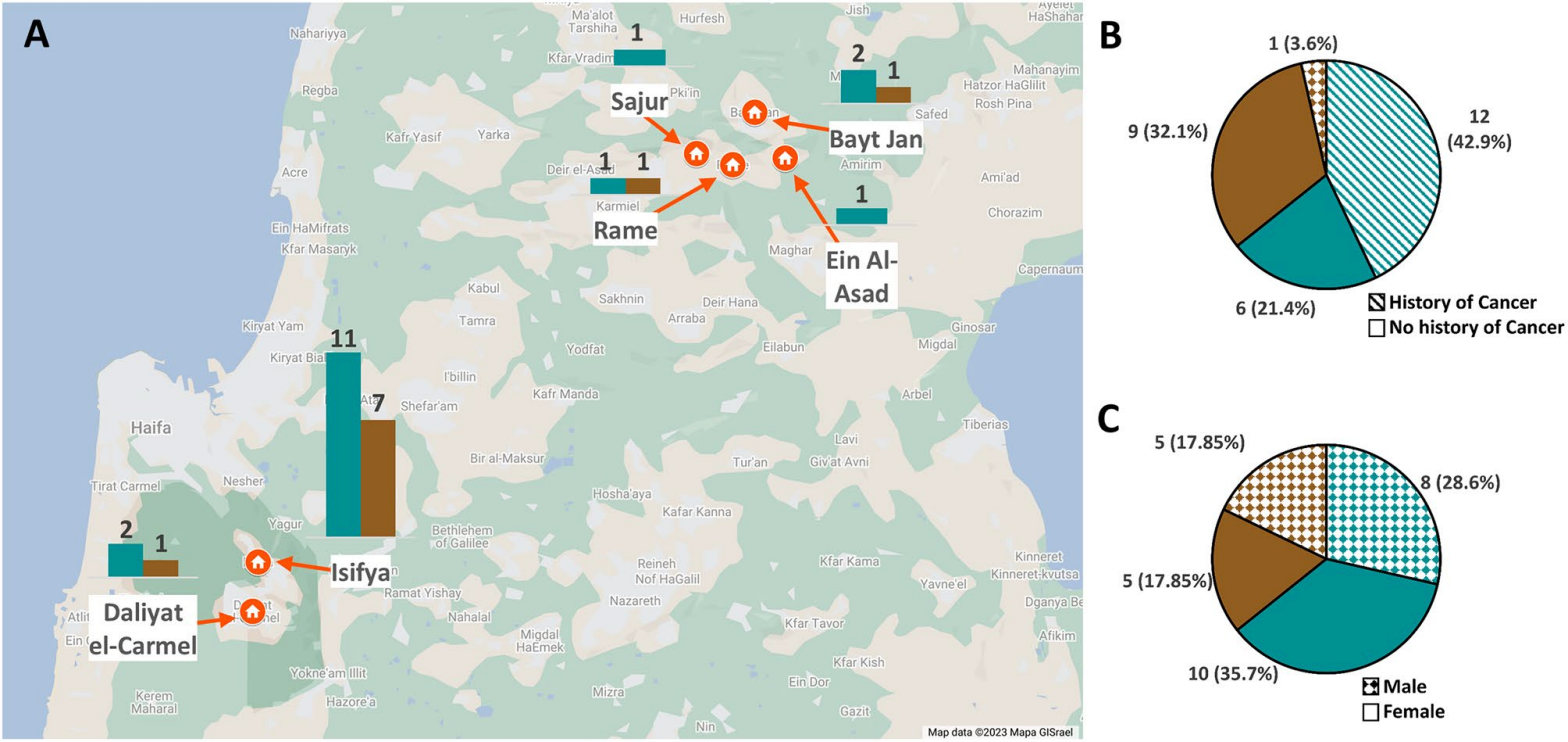
(**A**) Participants geographical distribution; shown a map of northern Israel acquired from Google Maps, showing the location of the 6 towns that samples were collected from and the number of participants (Lynch syndrome or non-Lynch syndrome individuals) from each town on bar-graphs. (**B**) Pie chart presenting cancer history distribution (presented by the filling) within LS and non-LS groups (presented by the color). (**C**) Pie chart presenting gender distribution (presented by the filling) within LS and non-LS groups (presented by the color).

Our cohort consisted of 28 Druze individuals (54% females) (Fig. 1C) from a limited number of Druze communities in northern Israel (Fig. 1A). Of them, 18 participants were LS patients, and 10 were non-LS participants, family members of the LS patients some residing in the same household. Participants were evaluated for body mass index (BMI), cancer history, and medication intake (Fig. 1, Table S1). The median age of participants was 56 (range 23–86) and their median BMI was 26.8 (range 19.5–35.4). Among 18 LS participants 12 (66.7%) had a history of cancer, while one of the 10 non-LS participants had a history of cancer diagnosis 23 years prior to his recruitment to the study (Fig. 1B, Table 1). Within 18 LS participants, 10 (55.6%) had a history of cancer of the gastrointestinal tract (Table 1). Within the LS participants and history of cancer, 7 (58.3%) were diagnosed before the age of 50 (Table 1).

**Table 1:** Clinical features of history of cancer in Lynch participants.

		n	%
Lynch participants (n = 18)	History of cancer	12	66.7
Lynch participants with history of cancer (n = 12)	History of Lynch related cancer	10	83.3
	Age at diagnosis under 50	7	58.3

**Figure 2:**
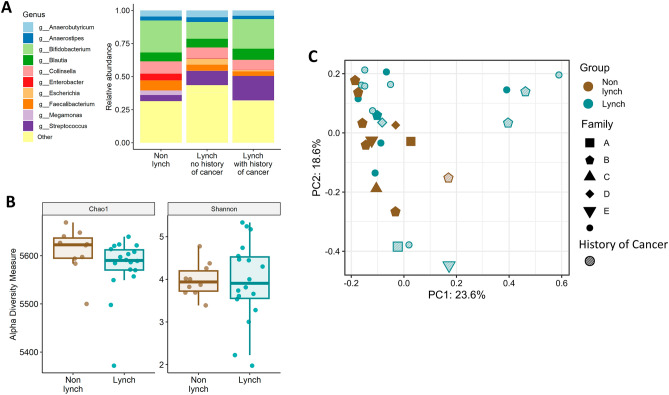
(**A**) Ten most abundant genera relative abundances presented in three different groups, non- lynch syndrome (left), lynch syndrome without (middle) or with (right) cancer in their medical history. (**B**) Chao1 (left) and Shannon (right) indices for α-diversity of the non-lynch syndrome group compared to the lynch syndrome group, data represent the median (line in box), IQR (box), and minimum/maximum (whiskers). (**C**) Bray–Curtis PCoA of samples’ β-diversity, each point presents one individual’s sample, lynch syndrome status is indicated by the color of the point while their cancer history is indicated by the shape of the point.

Shotgun metagenomic sequencing was performed on each of the samples with a mean library size of 57,473,662 reads (range 2,978,122–141,078,058). The ten most abundant genera analyzed from the metagenomics data, are presented in Fig. 2A. The bacterial differential abundance comparison between the LS groups (with or without cancer history) and the non-LS participants did not yield statistically significant results; however, some trends were observed. The *Streptococcus* genus was enriched in LS groups compared to non-LS participants. *Faecalibacterium* was less enriched in both LS groups compared to the non-LS participants. Additionally, *Escherichia* genus was also enriched in both LS groups compared to the non-LS participants, however this enrichment was higher in the LS group with no cancer history compared to LS with cancer history. Interestingly, *Bifidobacterium* genus was enriched both in the non-LS participants and in the LS group with cancer history but not in LS with no cancer history.

The samples’ α-diversities were measured using the Chao1 (sample’s richness) and Shannon (sample’s richness and evenness) indices. We found no significant differences in α-diversity between LS and non-LS participants, though the LS group showed higher variance (Fig. 2B). β-diversity (Bray–Curtis) PCoA analysis represents similarities and dissimilarities between samples. While some samples have clustered closer to each other than the rest of the samples these clusters did not reveal a clear difference between the groups (Fig. 2C, Fig. S1).

**Figure 3:**
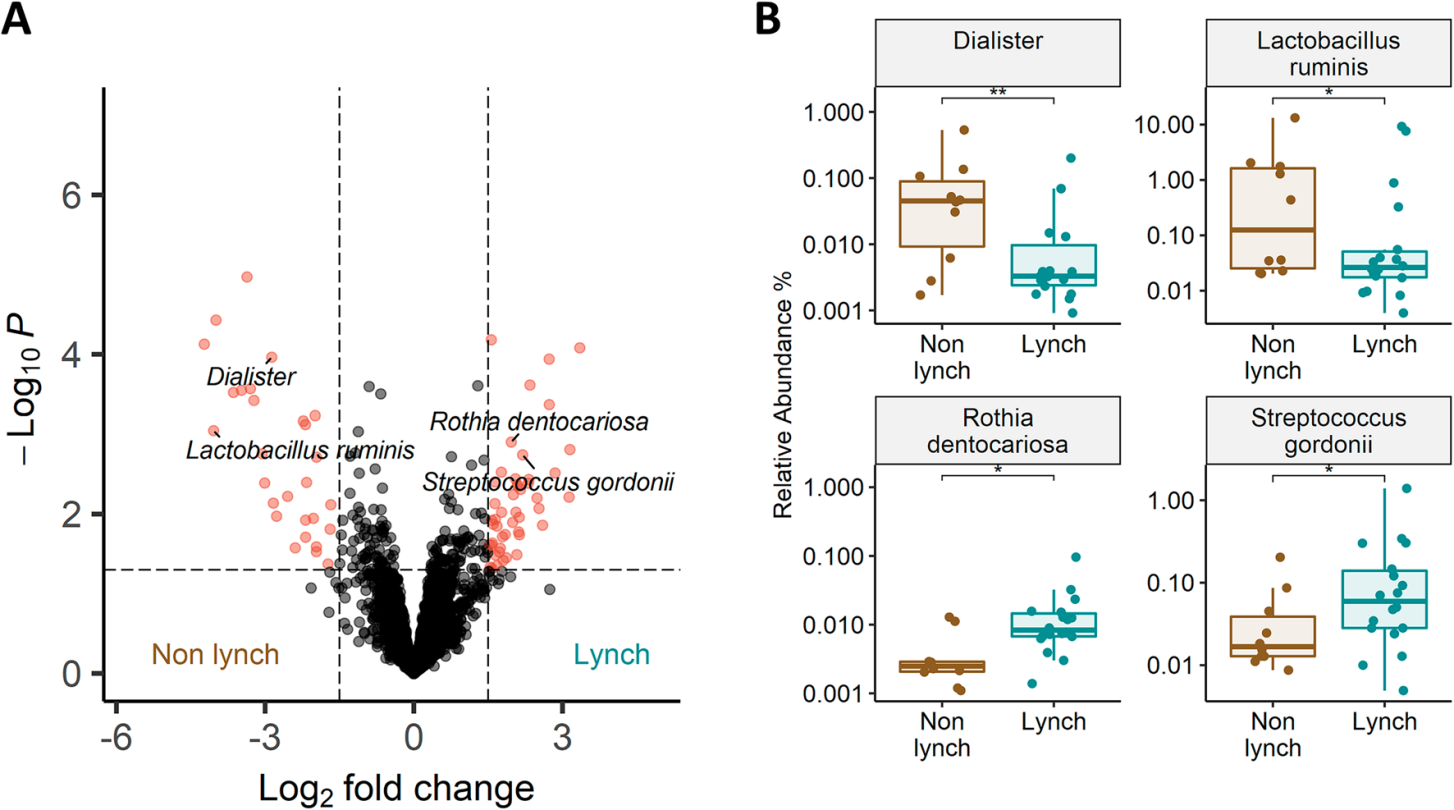
(**A**) Volcano plot of differential bacterial taxonomic units' abundance, between samples from LS participants and samples from non-LS participants. Red dots indicate differentially abundant bacteria with a p-value < 0.05 and log2 fold change > 1.5 and < − 1.5, respectively (detected by the DeSeq2 algorithm (Wald test, p < 0.05)). (**B**) Boxplots showing differential abundances of four bacterial taxonomic units for LS participants compared to non-LS participant, data represent the median (line in box), IQR (box), and minimum/maximum (whiskers) (Wald test, *p < 0.05, **p < 0.01).

Specific genera and species associated with LS were identified using DEseq2 (Wald test, alpha < 0.05 FDR), controlling for age, sex, and BMI groups. Analysis of the whole microbiota has revealed several genera and specific species, significantly altered between the groups, as shown in Fig. 3. LS participants had higher relative abundances of five *Streptococcus* species*, Enterococcus* genus, *Rothia dentocariosa*, *Rothia mucilaginosa*, and *Akkermansia muciniphila*, and lower abundances of *Intestinibaculum porci*, *Megamonas funiformis*, *Dialister* genus, as well as two *Dialister* species—*Dialister hominis* and *Dialister massiliensis*. Intriguingly, *Lactobacillus delbrueckii* was at higher relative abundance in LS participants relative to non-LS controls, while another Lactobacilli species, *Lactobacillus ruminis*, was lower (Fig. 3, Table S1).

**Figure 4:**
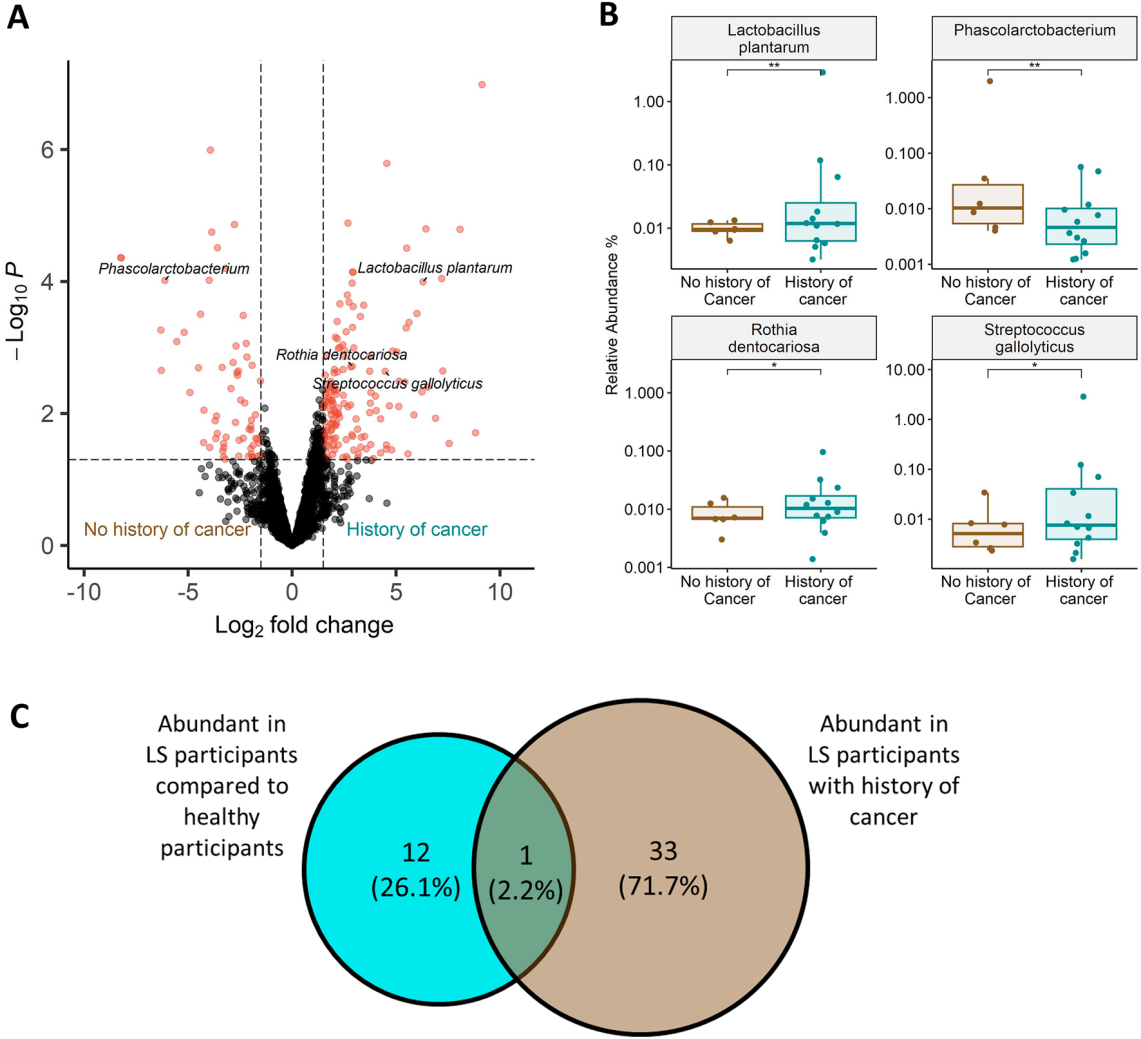
(**A**) Volcano plot of differential bacterial operational taxonomic unit abundance, between samples from LS participants with and without history cancer. Red dots indicate differentially abundant bacteria with a p-value < 0.05 and log2 fold change > 1.5 and < − 1.5, respectively (detected by the DeSeq2 algorithm (Wald test, p < 0.05)). (**B**) Boxplots showing differential abundances of four bacterial taxonomic units comparing LS participants depending on cancer history status within, data represent the median (line in box), IQR (box), and minimum/maximum (whiskers) (Wald test, *p < 0.05, **p < 0.01). (**C**) Venn diagram showing the number and percentages of bacterial operational taxonomic units enriched in LS participants, enriched in LS with a history of cancer, and enriched in both groups.

Next, we examined the correlations between microbiome composition and cancer history within the LS groups. Within all LS participants, specific genera species associated with history of cancer were identified using DEseq2 (Wald test, alpha < 0.05 FDR), controlling for age, sex, and BMI. LS participants with cancer history had higher relative abundances of seven *Streptococcus* species, *Enterococcus faecalis*, *Rothia dentocariosa*, and species of the *Lactobacillus* genus. Additionally, LS participants with cancer history had lower relative abundances of *Victivallales bacterium*, a specific *Actinomyces* species, *Propionibacterium acidifaciens*, *Alistipes finegoldii*, as well as *Phascolarctobacterium faecium* and *Phascolarctobacterium succinatutens* (Fig. 4, Table S1).

To further validate that the differences identified regarding bacterial taxonomic units, significantly altered in LS vs. non-LS participants, were not driven by the LS participants’ history of cancer, we searched for differences in microbiome composition within the LS groups (with and without history of cancer). Only one bacterial species, *Rothia dentocariosa*, was altered both when comparing LS vs. non-LS participants *and* when comparing LS participants based on their cancer history (Fig. 4C).

**Figure 5:**
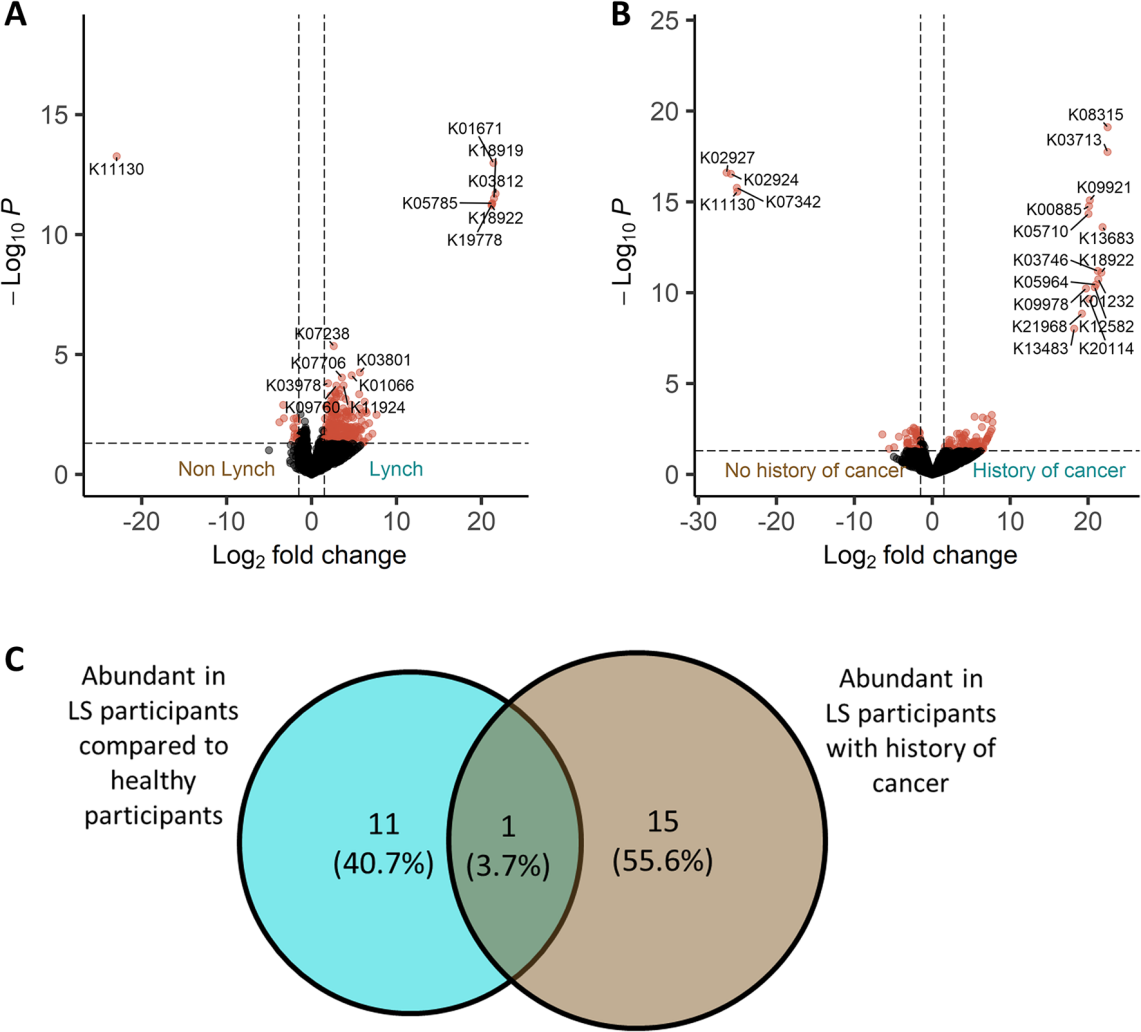
(**A**) Volcano plot of differential KEGG orthologs (KOs) abundance, between samples from LS participants and samples from non-LS participants. Red dots indicate differentially abundant bacteria with a p-value < 0.05 and log2 fold change > 1.5 and < − 1.5, respectively (detected by the DeSeq2 algorithm (Wald test, p < 0.05)). (**B**) Volcano plot of differential KOs abundance, between samples from LS participants with and without history cancer. Red dots indicate differentially abundant bacteria with a p-value < 0.05 and log2 fold change > 1.5 and < − 1.5, respectively (detected by the DeSeq2 algorithm (Wald test, p < 0.05)). (**C**) Venn diagram showing the number and percentages of KOs enriched in LS participants, enriched in LS with a history of cancer, and enriched in both groups.

On the functional level, we compared functional KEGG orthologs (KOs) abundances between LS and non-LS participants, and cancer history within the LS groups. In total, 14 pathways differed significantly between LS and non-LS participants (DEseq2, Wald test, alpha < 0.05 FDR). Only ‘K11130: H/ACA ribonucleoprotein complex subunit 3’ was associated with non-LS samples, while KOs associated with LS with the highest foldchange compared to non-LS were ‘K01671: sulfofructosephosphate aldolase’, ‘K03812: ribosome modulation factor’, ‘K18919: protein HokC/D’, ‘K05785: transcriptional antiterminator RfaH’, ‘K19778: acid stress chaperone HdeB’, and ‘K18922: protein HokE’ (Fig. 5A, Table S1). Among all LS participants, we found 19 specific KOs associated with history of cancer (Fig. 5B, Table S1). The results revealed one KO that had been associated with both LS and an history of cancer—‘K18922: protein HokE’, and one KO that had been associated with both having no history of cancer and being non-LS—‘K11130: H/ACA ribonucleoprotein complex subunit 3’ (Fig. 5C).

## Discussion

Host genetics can modulate the gut microbiome composition^27^. Specifically, LS is characterized by a mutation in mismatch repair genes, which was shown to affect mucosal immunity^28^. Notably, the gut microbiome resides in an intricate relationship with the mucosal immune system and as a result, its composition can be altered^29^. LS predisposes patients to cancer, however not all patients develop the disease. Moreover, LS patients, even with the same mutation, display phenotypic variability in age of cancer diagnosis and cancer location. The gut microbiome has emerged as a major regulator of human health. Alterations in the microbiome composition were demonstrated in a plethora of diseases including in CRC and other cancer types.

Altogether, these led us to hypothesize that the gut microbiome is altered in LS patients, and further may be altered based on their cancer history. We chose to test this hypothesis in a genetically and culturally homogenous community that shares similar environmental factors and resides in close proximity, geographically. Our cohort included LS individuals and their family members as non-LS controls, all within the Druze community of northern Israel.

We find specific operational taxonomic units (OTUs) enriched in LS patients while other OTUs decrease in abundance. The *Dialister* genus, which was decreased in LS, was recently shown to be over-represented in patients with advanced lung cancer, where it was correlated with weak response to immunotherapy^30^. On the other hand, *Rothia dentocariosa*, which we found to be increased in LS, was also increased in stool of lung and hematological cancer patients and is negatively correlated with chemotherapy efficacy^31^. *Lactobacillus ruminis*, decreased in LS, was demonstrated as a pro-inflammatory bacteria^32^, and *Streptococcus gordonii*, increased in LS, was associated with tumor tissues and not healthy tissues of oral squamous cell carcinoma^33^. Notably, *S. gordonii* was associated in several studies with *Fusobacterium nucleatum*—a well-studied bacteria in association with malignancy and aggressiveness of CRC as well as other tumors^8,12,34–37^. Altogether, this implies that the types of bacteria that we identified in LS individuals have been previously reported to be associated with cancer, cancer aggressiveness and potential response to therapy.

Within LS participants we find specific OTUs enriched or decreased in abundance in participants with cancer history compared to those with no cancer history. Among the bacteria which were enriched in LS participants without cancer history we find *Phascolarctobacterium*, a bacterial genus previously associated with lower levels of C-reactive protein (CRP) (a common measure of inflammation)^38^. We also find enrichment of *Lactobacillus plantarum,* which was extensively studied in various diseases and effects on the host^39,40^, and specifically was shown to exert anti-inflammatory effects, inducing IL-10 and alleviating Inflammatory bowel disease (IBD)^41^. Notably, these bacteria are associated with low inflammatory states, and potential anti-inflammatory functions, both align with their presence in LS participants without cancer history, and can be potentially targeted, in the future, as biomarkers for LS participants with lower probability of developing cancer.

In parallel, *Streptococcus gallolyticus* and *Rothia dentocariosa,* which were enriched in LS participants with cancer history, were also shown in several other studies to be enriched in cancer patients. Specifically, *S. gallolyticus* was shown to have higher relative abundances in CRC patients and positively correlated with higher probability of adenoma or adenocarcinoma^42^. Therefore, these bacteria, which are persistently associated with cancer, can be future candidates for both cancer diagnostics biomarkers and therapeutic targets. For example, future studies may evaluate, experimentally, whether their elimination can reduce cancer initiation and progression. Overall comparison between all analyses (LS vs. non-LS, and cancer vs. no cancer within the LS patients) revealed distinct microbial populations with only one bacteria overlapping in all analyses (Fig. 4C). This bacteria is *Rothia dentocariosa* which was enriched in both LS and in LS with cancer history relative to controls. Thus, *R. dentocariosa* is a specifically interesting bacteria for future studies on LS, and for experimental validation, examining its possible functions in cancer initiation, uniquely in LS patients.

Our analysis of functional KEGG ortholog abundances patterns between participants with LS and non-LS revealed a difference in 14 pathways between LS and non-LS participants. Particularly, specific KOs demonstrated distinct associations with either LS or non-LS samples. For instance, ‘K18922: protein HokE’ displayed a dual association with both LS and a history of cancer. These observations provide valuable insights into potential functional disparities related to genetic pathways, Lynch syndrome, and cancer history within our study cohort.

Since only one bacteria and two KOs overlapped, this emphasizes that the enriched bacteria and KOs identified in LS patients are not due to overrepresentation of cancer history within the LS group. To note, the overall alpha and beta-diversities were not significantly different between neither of the groups, suggesting that the microbial uniqueness can only be defined on the OTU levels and not when analyzing the whole microbiome diversity. It is possible that a larger cohort would enable detection of differences in alpha and beta-diversities as well.

To the best of our knowledge this study is unique in multiple aspects: (1) We focused on one specific LS mutation, a nonsense mutation in the MSH2 gene. Other studies included either mutations in several genes or multiple (different) mutations in the same gene. (2) We studied a presumably uniform, population—the Druze community of the north of Israel. (3) Our study compared between family members, including household members, with presumably similar genetic background as well as similar habits, diets etc. Nonetheless, some family members albeit harboring the same MSH2 mutation, differed in their cancer history. (4) The depth of the sequencing we performed was extremely large (30 GB) enabling high-resolution analyses.

Our study, focused on the fecal microbiome in individuals with Lynch syndrome, is characterized by a relatively small cohort of 28 participants, and by inclusion of diverse cancer types. The cross-sectional design precludes the exploration of dynamic microbiome changes over time, an avenue for future research. Furthermore, we excluded individuals who had undergone antibiotic treatment within six weeks prior to sample collection. However, we are aware of the potential of long-term impact of antibiotic treatment on microbiome composition, which is an additional consideration when interpreting our findings. Lastly, the study’s exclusive focus on the Druze population in Israel may restrict the generalizability of our findings. We acknowledge the preliminary nature of our study, highlighting the need for future investigations to address these limitations, refine our understanding, and extend our findings to broader contexts.

Our results offer insights into defined and significant microbiome differences, unique to the LS population, as well as specific microbiome alterations in LS patients with history of cancer. This was made possible due to the uniqueness of the Druze community, with one founder LS causing mutation. We focused on families and households, with or without LS mutations, living in identical or adjacent geographical regions with similar environmental conditions. This gave us the opportunity to study, categorically, the microbiome changes caused, most probably, by the LS-mutation genetic background. The defined OTU differences, which we identified, can be examined mechanistically in future studies, and may provide novel microbiome-based targets for diagnostics and therapeutic interventions.

### Limitations of the study

The relatively small sample size of our cohort, and the inclusion of patients who developed several types of cancer (albeit predominantly LS-associated), and underwent several types of treatments (i.e. chemotherapy, surgery, immunotherapy), limit the statistical power of this study. For example, surgery alone might have a direct effect on the microbiome composition. However, the main goal of this study was to focus on a homogenous community in regards to the LS mutation, culture, and geographical living location, and this led to this compromise in the participants’ cancer types and treatments, calling for further studies in such homogenous populations, with larger cohorts. Lastly, aspirin, which was demonstrated as a chemoprevention agent for LS patients, and is used in medical practice^43,44^, was not documented in this study since the Israeli Gastroenterology Association is currently not endorsing its routine use for chemoprevention in LS.

## Methods

### Inclusion criteria

All consecutive Druze individuals over 18 years of age, found to carry a MSH2 gene mutation either probands or non-LS family members were offered to participate in the study.

All LS participants were genotyped as harboring a germline pathogenic mutation in MSH2. Non-LS biological family members were genotyped to be defined as non-LS participants. One biological family member was defined as obligatory negative (i.e., the subject’s biological relation to LS family was confirmed as a genotyped negative parent). Non-biological family members were defined as non-LS participants based on family and personal medical history.

### Exclusion criteria

Patients were excluded from the study if they had recent (6 weeks) antibiotic treatment, recent surgery (6 weeks), or unable or unwilling to sign in form consent.

### Standard protocol approvals, registration and patient consents

This study was approved by the local institutional review board of the Rambam Health Care Campus and was held in accordance with relevant guidelines and regulations (Helsinki 0129-13). Written informed consent was obtained from all included subjects prior to enrollment in accordance with the Declaration of Helsinki.

### Sample collection and processing

Fecal samples were self-collected by LS and non-Lynch individuals in stool sample cups. Fecal samples were stored in − 80 °C within two hours of collection or, otherwise, stored in − 20 °C until they were delivered to the hospital research team, where they were stored in − 80 °C. Each fecal sample was sub-sampled without thawing the whole fecal sample. Sub-samples were heat inactivated at 65 °C for 20 min. DNA was extracted from all samples using the PureLink Microbiome DNA purification kit (catalog number: A29790), according to the manufacturer’s instructions with matching negative controls. Samples were collected 4 weeks or more after the subjects’ latest endoscopic colonoscopies.

### Library preparation

DNA samples underwent quality control by Qubit fluorescence analysis to determine concentration of DNA for downstream analysis (ThermoFisher, Cat. Q32850). Libraries were prepared for the samples and for the library preparation controls using the Illumina Tagmentation DNA prep streamlined library preparation protocol according to manufacturer’s instructions with a minimum of 50 ng of DNA starting mass and 8 cycles of PCR enrichment, ending with a fragment size of 550 bp. IDT for Illumina DNA/RNA UD indexes and Nextera DNA CD indexes were used (Illumina IDT, Cat. 20027213; Illumina Nextera, Cat. 20018708).

### Sequencing

All libraries were diluted to 15 pM in 96-plex pools and validated on 100-cycle paired-ends read Miseq V2 runs (Illumina, Cat. MS-102-2002), before shipping to the US at 4 nM for sequencing on the Novaseq 6000 in S4 mode at 96-plex in a 300-cycle paired-end reads run, with an estimated read depth of 30 Gbp per sample (Illumina, Cat. 20028312). Final loading concentration of 600 pM. All sequencing runs were performed with a spike-in of 1% PhiX control library V3 (Illumina, Cat. FC-110-3001).

### Taxonomic profiling

Sequence data quality control, including removal of human reads, was performed using KneadData^45^ (version 0.12.0) with default parameters. Reads passing the quality control were mapped to the NCBI nucleotide database using Centrifuge. Taxonomic annotations for each read were obtained using the least common ancestor algorithm, and then summarized across all reads to create counts per taxon. Raw counts were normalized using total sum scaling. Raw counts were normalized to percentages for relative abundance. Taxonomic data was filtered for taxa with a minimum of 0.01% relative abundance across all samples, and detection in at least 20% of the samples. Library prep controls were used to assess contaminations present in the data.

Gene function analysis of the samples was conducted using HUMAnN 3.8^45^ with the default settings on samples evenly rarified to 10 million reads. The resulting UniRef90 gene families were transformed into KO groups^46^ using the ‘humann_regroup_table’ tool from the HUMAnN software. KO groups were then refined to include only community-level data. Data was then transformed by centered-log ratio.

### Statistics and bioinformatics

Statistical analysis of Sequenced data was initially performed using MicrobiomeAnalyst^47^ followed by comprehensive analysis using R packages: Phyloseq^48^, Vegan^49^ and DESeq2^50^. Differences in microbial taxa and functional profiles were assessed by differential abundance analyses using DESeq2^50^. Alpha diversity indices (Chao1 and Shannon) were compared using the Kruskal–Wallis rank sum test. Beta diversity distance matrices (Bray–Curtis) were compared using the vegan package’s function ADONIS, a multivariate ANOVA based on dissimilarity tests and visualized using PCoA (taxonomy). Results were visualized using the R packages ggplot2^51^, EnhancedVolcano^52^ with palettes from the MetBrewer package^53^.

### Supplementary Information

Supplementary Figure S1.

Supplementary Tables.

## Data Availability

Raw metagenomic data are deposited to the NCBI Sequence Read Archive (SRA) database and assigned BioProject accession no. PRJNA939026.
