# Supplementary material for: Gut microbial signatures are associated with Lynch syndrome (LS) and cancer history in Druze communities in Israel
Source: Sci Rep. 2023 Nov 24;13:20677. doi: 10.1038/s41598-023-47723-3 (PMC10673896; doi:10.1038/s41598-023-47723-3)
Supplement: Supplementary file 1 — Supplementary Figure S1. [file 41598_2023_47723_MOESM1_ESM.docx]

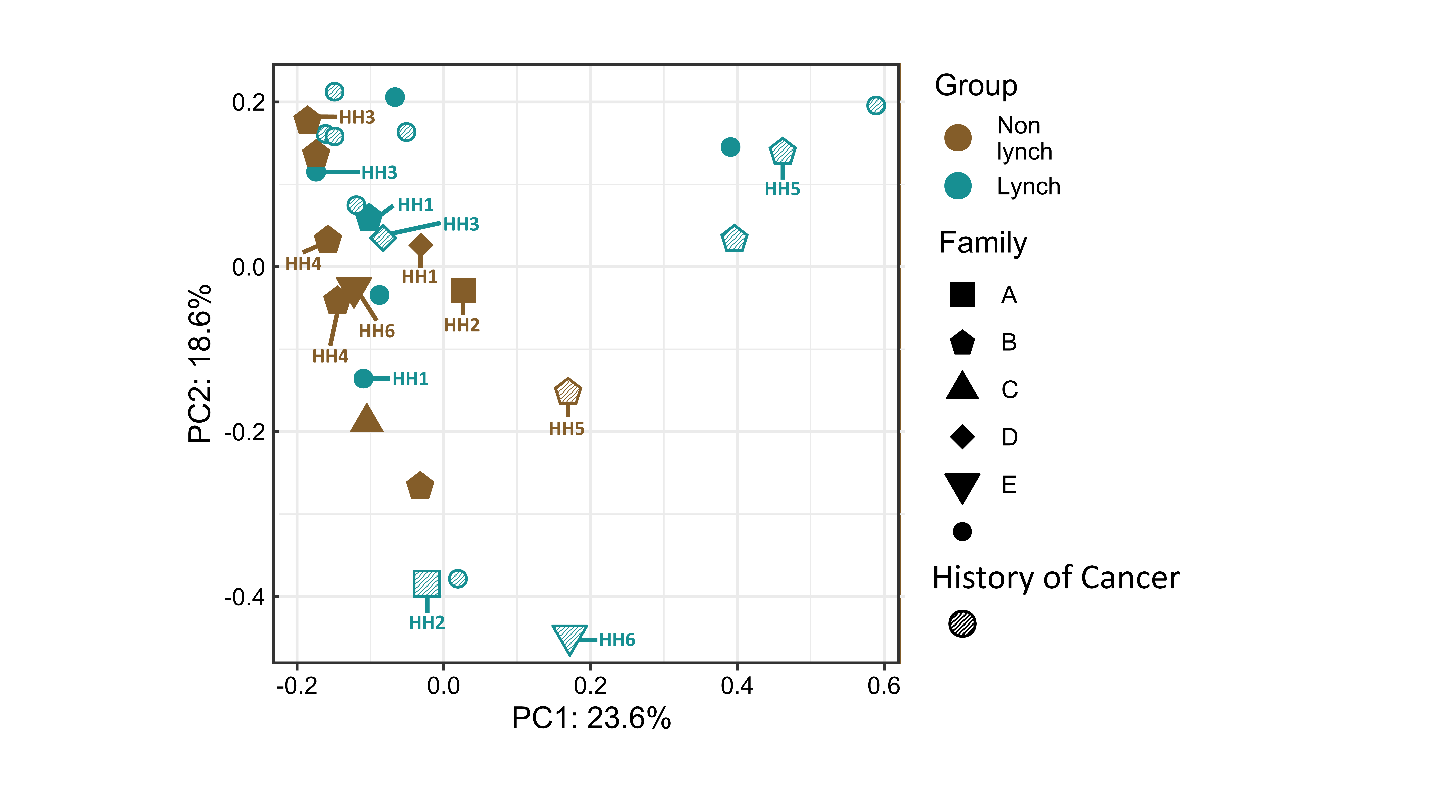


**Figure S1**. Bray-Curtis PCoA of samples’ β-diversity, each point presents one individual’s sample, lynch syndrome status is indicated by the color of the point while their cancer history is indicated by the shape of the point and their household is noted.
